# Nonalcoholic fatty liver disease and increased risk of 1-year all-cause and cardiac hospital readmissions in elderly patients admitted for acute heart failure

**DOI:** 10.1371/journal.pone.0173398

**Published:** 2017-03-13

**Authors:** Filippo Valbusa, Stefano Bonapace, Davide Agnoletti, Luca Scala, Cristina Grillo, Pietro Arduini, Emanuela Turcato, Alessandro Mantovani, Giacomo Zoppini, Guido Arcaro, Christopher Byrne, Giovanni Targher

**Affiliations:** 1 Division of General Medicine “Sacro Cuore” Hospital, Negrar, Verona, Italy; 2 Division of Cardiology, “Sacro Cuore” Hospital, Negrar, Verona, Italy; 3 Division of Geriatrics, “Sacro Cuore” Hospital, Negrar, Verona, Italy; 4 Section of Endocrinology, Diabetes and Metabolism, Department of Medicine, University and Azienda Ospedaliera Universitaria Integrata of Verona, Verona, Italy; 5 Nutrition and Metabolism, Faculty of Medicine, University of Southampton, Southampton, United Kingdom; 6 Southampton NIHR Biomedical Research Centre, University Hospital Southampton, Southampton, United Kingdom; Bambino Gesù Children's Hospital, ITALY

## Abstract

Nonalcoholic fatty liver disease (NAFLD) is an emerging risk factor for heart failure (HF). Although some progress has been made in improving survival among patients admitted for HF, the rates of hospital readmissions and the related costs continue to rise dramatically. We sought to examine whether NAFLD and its severity (diagnosed at hospital admission) was independently associated with a higher risk of 1-year all-cause and cardiac re-hospitalization in patients admitted for acute HF. We studied 212 elderly patients who were consecutively admitted with acute HF to the Hospital of Negrar (Verona) over a 1-year period. Diagnosis of NAFLD was based on ultrasonography, whereas the severity of advanced NAFLD fibrosis was based on the fibrosis (FIB)-4 score and other non-invasive fibrosis scores. Patients with acute myocardial infarction, severe valvular heart diseases, end-stage renal disease, cancer, known liver diseases or decompensated cirrhosis were excluded. Cox regression was used to estimate hazard ratios (HR) for the associations between NAFLD and the outcome(s) of interest. The cumulative rate of 1-year all-cause re-hospitalizations was 46.7% (*n* = 99, mainly due to cardiac causes). Patients with NAFLD (*n* = 109; 51.4%) had remarkably higher 1-year all-cause and cardiac re-hospitalization rates compared with their counterparts without NAFLD. Both event rates were particularly increased in those with advanced NAFLD fibrosis. NAFLD was associated with a 5-fold increased risk of 1-year all-cause re-hospitalization (adjusted-hazard ratio 5.05, 95% confidence intervals 2.78–9.10, *p*<0.0001) after adjustment for established risk factors and potential confounders. Similar results were found for 1-year cardiac re-hospitalization (adjusted-hazard ratio 8.05, 95% confidence intervals 3.77–15.8, *p*<0.0001). In conclusion, NAFLD and its severity were strongly and independently associated with an increased risk of 1-year all-cause and cardiac re-hospitalization in elderly patients admitted with acute HF.

## Introduction

The prevalence of heart failure (HF) is high (≥10%) among persons aged 70 years or older and its incidence is rapidly increasing, due to better life expectancy [1,2]. To date, although some progress has been made in improving survival in hospitalized patients with acute HF, the rates of hospital readmissions are rising dramatically, especially in the elderly [2,3]. High readmission rates not only drive burgeoning health care costs but also suggest that management of HF is suboptimal [3]. Identifying novel predictors of hospital readmissions for HF in the elderly would facilitate better discharge planning and, perhaps, decrease readmission rates.

Nonalcoholic fatty liver disease (NAFLD) is a multisystem disease that affects many organ systems, including both the heart and the vasculature [4,5]. Growing evidence indicates that patients with NAFLD have early changes in cardiac substrate metabolism, producing functional, structural and arrhythmic consequences that are potentially linked to an increased risk of new-onset HF [6]. For example, some population-based studies reported a strong association between mildly elevated serum liver enzymes (a surrogate marker of NAFLD) and increased long-term risk of new-onset HF [7–9]. Additionally, the Coronary Artery Risk Development in Young Adults (CARDIA) Investigators recently found that computed tomography-diagnosed NAFLD was independently associated with subclinical myocardial remodeling and dysfunction, thus providing further insight into the potential link between NAFLD and HF [10].

These findings support the view that NAFLD is implicated in HF development and might be also a predictor of higher hospital readmissions for acute HF. We have previously explored the association between NAFLD and the risk of all-cause hospital readmissions in a small sample of elderly patients admitted for acute HF [11]. However, the sample size was much lower than that of the current study and no detailed information regarding the causes of hospital readmissions as well as the severity of hepatic fibrosis in patients with NAFLD was available [11].

Consequently, it remains currently uncertain whether NAFLD and its severity may independently predict 1-year hospital re-admissions after HF. Thus, the aim of this study was to examine whether ultrasound-diagnosed NAFLD and its severity–using the fibrosis (FIB)-4 score or other non-invasive markers of advanced NAFLD fibrosis–were associated with an increased risk of 1-year all-cause and cardiac re-hospitalization in patients admitted initially to the hospital with HF.

## Materials and methods

### Patients

We studied a cohort of patients consecutively admitted with a diagnosis of acute HF to the Divisions of General Medicine or Geriatrics at the ‘Sacro Cuore’ Hospital of Negrar (Verona) over the years 2013 and 2014 (*n* = 314). All patients were initially eligible for the study if they had a confirmed clinical diagnosis of acute HF (pre-existing or *de novo* HF). In agreement with the 2012 European Society of Cardiology guidelines [2], the clinical diagnosis of acute HF was based on the presence of typical signs and symptoms of acute HF, increased NT pro-brain natriuretic peptide (NT-proBNP) levels as well as radiographic findings of acute HF.

From the initial eligible cohort, we excluded 102 (32.5%) patients with: 1) acute myocardial infarction, end-stage kidney disease or malignancy (*n* = 11); 2) severe heart valve diseases or prior heart valve surgery (*n* = 22); 3) decompensated cirrhosis or other known causes of chronic liver diseases, including viral hepatitis and excessive alcohol consumption (defined as >30 g/day for men and >20 g/day of alcohol intake for women, respectively) (*n* = 34); and 4) those who died during the first hospital admission (in-hospital deaths; *n* = 35). As consequence of this selection, 212 (67.5%) elderly patients hospitalized for acute HF were included in the final analysis.

The local ethics committee/IRB of the ‘Sacro Cuore’ Hospital approved the study protocol, and all participants gave their written informed consent.

### Clinical and laboratory data

Body mass index (BMI) was measured as kilograms divided by the square of height in meters. Blood pressure was measured with a standard mercury sphygmomanometer after patient had been seated quietly for at least 5 min. Patients were considered to have hypertension if their blood pressure was ≥140/90 mmHg or if they were taking any anti-hypertensive drugs.

Serum levels of creatinine, liver enzymes (aspartate and alanine aminotransferases [AST and ALT] and gamma-glutamyltransferase [GGT]), electrolytes, complete blood count and other biochemical blood measurements were determined by standard laboratory procedures in the central Laboratory of the hospital for all patients. Plasma NT-proBNP measurements were determined using a chemiluminescent immunoassay method. Most patients had serum liver enzyme levels within the reference ranges in our Laboratory, which for serum GGT levels were 60 U/l for both sexes, and for aminotransferases were 10 to 40 U/l for women and 10 to 50 U/l for men, respectively. We also calculated the APRI index (AST to platelet ratio index), the fibrosis (FIB)-4 score (that includes age, serum aminotransferases and platelet count in its equation) and the NAFLD fibrosis score (that includes age, BMI, impaired fasting glycaemia/diabetes status, serum aminotransferases, albumin and platelet count in its equation) in patients with NAFLD as non-invasive markers of advanced hepatic fibrosis, using for the FIB-4 and NAFLD fibrosis scores the new cutoffs proposed for patients with NAFLD aged ≥65 years [12]. Glomerular filtration rate (eGFR_CKD-EPI_) was estimated using the Chronic Kidney Disease Epidemiology Collaboration (CKD-EPI) study equation [13].

Presence of coronary heart disease (CHD) was defined as a documented history of myocardial infarction, angina or coronary revascularization procedures. Chronic kidney disease (CKD) was defined as the presence of eGFR_CKD-EPI_ <60 ml/min/1.73 m^2^; measurements of albuminuria or proteinuria were not available. The diagnosis of persistent or permanent atrial fibrillation was made on the basis of medical history (from reviewing hospital and physician charts from all patients) and standard electrocardiograms. The presence of chronic obstructive pulmonary disease (COPD) was confirmed by reviewing medical records of the hospital, including diagnostic symptoms patterns, and results of lung function tests.

### Hepatic ultrasonography and conventional echocardiography

At baseline, experienced radiologists (blinded to the patients’ clinical details) performed hepatic ultrasonography for all patients. Hepatic steatosis was diagnosed based on characteristic ultrasonographic features, such as diffuse hyperechogenicity of the liver relative to the kidneys, ultrasound beam attenuation and poor visualization of the intra-hepatic vessel borders and diaphragm [14,15]. It is known that ultrasonography has a high sensitivity and specificity for detecting moderate and severe hepatic steatosis. However, its sensitivity is reduced when less than 20–30% of hepatocytes are steatotic [14].

Conventional trans-thoracic echocardiography, which was performed by experienced cardiologists (blinded to the patients’ clinical details), was used to measure left ventricular (LV) diameters and wall thicknesses according to international standard criteria [16]. LV end-diastolic and end-systolic volumes and ejection fraction at rest were measured at the apical 4-chamber and 2-chamber views (by modified Simpson rule) [16]. Echocardiographic measurements were available in the majority of our patients (*n* = 196, 92.5%).

### Statistical analyses

Data are presented as means±SD, medians and interquartile ranges or percentages. The primary outcome of the study was the first re-hospitalization at 1 year. Re-hospitalization data were obtained from either reviewing the patients’ hospital records or contacting the patients’ physician and the referring cardiologist or contacting patients directly.

Differences in baseline clinical and biochemical characteristics between patients stratified by 1-year re-hospitalization status at follow-up were tested with either the unpaired Student’s *t*-test (for normally distributed variables) or the Kruskal-Wallis test for non-normally distributed variables (*i*.*e*., serum liver enzymes, triglycerides, FIB-4 score, eGFR_CKD-EPI_ and NT-proBNP). The χ2 test was used to test for between-group differences among the categorical variables.

Univariate survival analysis was performed by the Kaplan-Meier analysis and the overall significance was calculated by the log-rank test. Cox regression analysis was used to examine the association between baseline NAFLD status and 1-year all-cause or cardiac re-hospitalization rates after adjustment for potential confounding variables. The model assumptions for the Cox proportional hazard regression models were checked by visual inspection of proportional hazard assumption, Schoenfeld’s residuals and covariance matrix. Four forced-entry Cox regression models were performed: an unadjusted model; a model adjusted for age, sex and hospital ward (General Medicine *vs*. Geriatrics) (model 1); a model adjusted for age, sex, hospital ward, past history of HF, obesity (i.e., BMI ≥30 kg/m^2^), diabetes, CHD, eGFR_CKD-EPI_, plasma NT-proBNP levels and LV-ejection fraction (model 2); and, finally, a regression model additionally adjusted for serum sodium and GGT concentrations (model 3). Covariates included in these multivariable regression models were chosen as potential confounding factors on the basis of their significance in univariable analyses or on the basis of their biologic plausibility. Results of Cox regression models were presented as hazard ratios (HR) and 95% confidence intervals (CI). *P* values <0.05 were considered statistically significant.

## Results

Overall, the patients included in the study had a mean age of 82±9 years, 59% had permanent/persistent atrial fibrillation, 37.8% had established diabetes, 35.4% had CKD, 33.5% had pre-existing CHD, 28% had a past history of HF, 18% had a LV-ejection fraction ≤40%, and 51.4% patients had NAFLD (defined as presence of fatty liver on ultrasonography among patients with no history of excessive alcohol consumption or other known causes of chronic liver disease).

In the whole cohort, the first all-cause re-hospitalizations at 1 year occurred in 99 (46.7%) patients. Overall, 78% (*n* = 77) of these re-hospitalizations were due to worsening HF and 22% to extra-cardiac causes (mainly due to respiratory and gastrointestinal diseases). The cumulative re-hospitalization rates were 11.3% (*n* = 24) at 1 month, 25.5% (*n* = 54) at 3 months, 36.8% (*n* = 78) at 6 months and 46.7% (*n* = 99) at 1 year.

Table 1 shows the baseline clinical and biochemical characteristics of patients stratified by re-hospitalization status during the follow-up. At baseline, patients who had been hospitalized during the follow-up were more likely to have pre-existing CHD and had higher plasma NT-proBNP and GGT levels and lower serum sodium levels than those not requiring re-hospitalization. Moreover, they were also more often treated with spironolactone, and tended to have (insignificantly) lower values of LV-ejection fraction and eGFR_CKD-EPI_. Notably, the prevalence of NAFLD and its severity (using the FIB-4 score) were remarkably greater in patients with re-hospitalization at follow-up than in those without.

**Table 1:** Baseline clinical and biochemical characteristics of hospitalized patients with acute HF stratified by 1-year all-cause re-hospitalization status at follow-up.

	Without re-hospitalization (*n* = 113)	With re-hospitalization (*n* = 99)	*p* value
Male sex (%)	44.2	46.5	0.75
Age (years)	82 ± 10	82 ± 8	0.92
Body weight (kg)	77 ± 23	75 ± 19	0.40
Body mass index (kg/m^2^)	27.8 ± 6	26.8 ± 6	0.35
Heart rate (bpm)	85 ± 22	82 ± 20	0.31
Systolic blood pressure (mmHg)	132 ± 21	132 ± 23	0.87
Diastolic blood pressure (mmHg)	75 ± 12	76 ± 12	0.75
Pulse pressure (mmHg)	57 ± 17	56 ± 19	0.66
Sodium (mmol/l)	138 ± 6	136 ± 5	<0.05
Potassium (mmol/l)	4.2 ± 0.5	4.2 ± 0.5	0.83
Hemoglobin (g/dl)	12.2 ± 2	11.9 ± 2	0.26
White blood cell count (x 10^9^/l)	8.03 ± 3	8.16 ± 3	0.76
Platelet count (x 10^9^/l)	226 ± 73	222 ± 84	0.72
eGFR_CKD-EPI_ (ml/min/1.73 m^2^)	55.1 ± 22	50.2 ± 22	0.09
GGT (U/l)	45 (24–81)	49 (24–96)	<0.05
AST (U/l)	23 (18–31)	25 (19–33)	0.07
ALT (U/l)	18 (12–26)	20 (13–32)	0.15
AST/ALT ratio	1.44 ± 0.8	1.28 ± 0.6	0.08
NT-proBNP (pg/ml)	579 (312–1018)	761 (400–1456)	<0.05
Total cholesterol (mmol/l)	3.72 ± 0.9	3.68 ± 0.9	0.81
Triglycerides (mmol/l)	0.99 (0.8–1.3)	0.98 (0.8–1.3)	0.74
LV-ejection fraction (%)	49.7 ± 14	46.2 ± 13	0.10
LV-ejection fraction ≤40% (%)	14.9	23.2	0.14
Diabetes (%)	37.2	38.4	0.86
Chronic obstructive pulmonary disease (%)	15.9	20.2	0.42
CHD (%)	24.8	43.4	<0.005
Stroke (%)	4.4	7.1	0.42
Pacemaker or ICD (%)	15.9	26.3	0.07
Atrial fibrillation (%)	57.5	60.6	0.65
Chronic kidney disease (%)	38.0	33.3	0.53
ACE-inhibitors/ARB users (%)	59.8	51.5	0.23
Furosemide users (%)	98.2	98.0	0.85
Spironolactone users (%)	29.5	43.4	<0.05
Beta-blocker users (%)	60.7	70.7	0.14
Digoxin users (%)	11.6	11.1	0.91
Amiodarone users (%)	2.7	3.0	0.89
Antiplatelet drug users (%)	36.6	49.5	0.09
Oral anticoagulant users (%)	39.3	39.4	0.99
Statin users (%)	22.3	25.3	0.62
Hospital stay (days)	13.6 ± 7	13.1 ± 6	0.62
Geriatric ward (%)	47.0	34.3	0.07
NAFLD (%)	31.9	73.7	<0.0001
FIB-4 <2 (normal)*	60.0	47.9	<0.0001
FIB-4 2–2.67 (intermediate)	22.9	15.1	
FIB-4 >2.67 (high)	17.1	37.0	

Sample size, *n* = 212. Data are expressed as means ± SD, medians (IQR) or relative proportions.

Note: Measurements of plasma NT-proBNP and LV-ejection fraction were available in 206 and 196 patients, respectively.

*The FIB-4 score was calculated only in patients with NAFLD.

Abbreviations: ARB, angiotensin receptor blocker; ALT, alanine aminotransferase; AST, aspartate aminotransferase; CHD, coronary heart disease; eGFR_CKD-EPI_, estimated glomerular filtration rate (as estimated by the CKD-EPI equation); FIB-4, fibrosis-4 score; GGT, gamma-glutamyltransferase; LV, left ventricular; NAFLD, nonalcoholic fatty liver disease; NT-proBNP, NT pro-brain natriuretic peptide.

At baseline, the two groups of patients did not significantly differ in terms of age, sex, BMI, heart rate, blood pressure, complete blood count, plasma lipids, pre-existing diabetes and other comorbid conditions (CKD, COPD and atrial fibrillation), nor in terms of the hospital length of stay and current use of many ‘cardiovascular’ medications (including the use of lipid-lowering drugs, ACE-inhibitors, angiotensin receptor blockers, beta-blockers, furosemide, digitalis, amiodarone, antiplatelet agents or anticoagulants).

S1 Table shows the clinical and biochemical data of the patients stratified by the hospital ward. Patients admitted to the Geriatric ward were older and had a lower body weight and a longer hospital stay than those admitted to the General Medicine ward. The two groups of patients did not differ significantly in terms of most of other clinical and biochemical data, except for a higher proportion of patients admitted to the General Medicine ward who were treated with spironolactone or antiplatelet agents and who had established diabetes or NAFLD.

When the whole sample of patients was stratified by NAFLD status at baseline, patients with NAFLD (*n* = 109) were more likely to be male (56% *vs*. 34%), younger (80±9 *vs*. 84±9 years) and had higher BMI (28.4±7 *vs*. 25.8±5 kg/m^2^), higher serum triglycerides (1.19±0.5 *vs*. 0.95±0.4 mmol/l) and lower values of AST/ALT ratio and eGFR_CKD-EPI_ compared to those without NAFLD (*n* = 103). On the contrary, the two groups of patients did not differ significantly in terms of most of the other clinical and biochemical data, including plasma NT-proBNP levels and LV-ejection fraction (data not shown).

**Fig 1:**
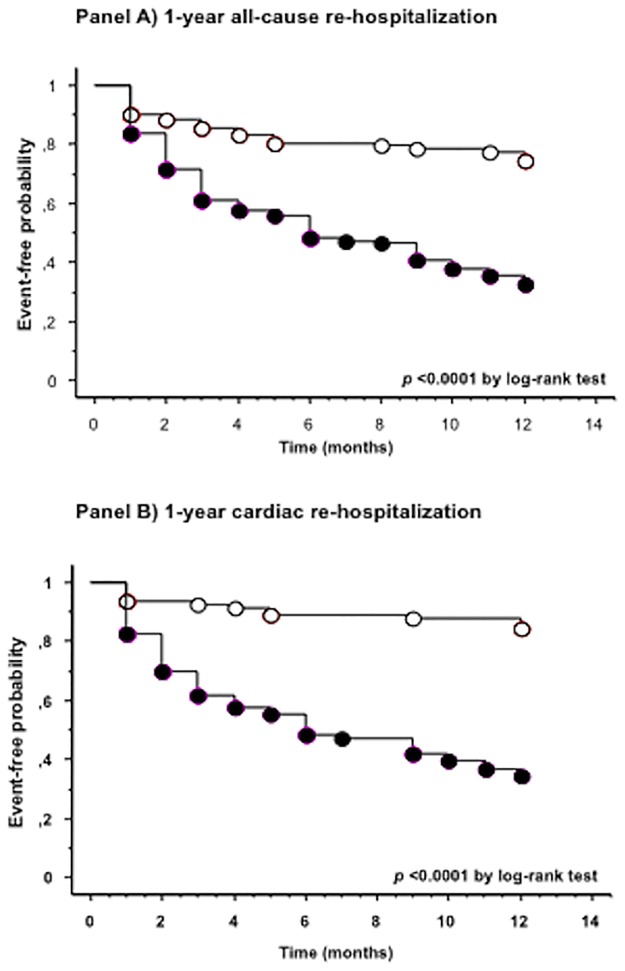
Kaplan-Meier curves. Rates of 1-year all-cause (panel A) or cardiac (panel B) re-hospitalization in hospitalized patients with acute HF stratified by their ultrasound-diagnosed NAFLD status at baseline. Patients with NAFLD: closed circles; patients without NAFLD: open circles. *P*<0.0001 for the difference by the log-rank test.

The cumulative proportions of patients with 1-year all-cause or cardiac re-hospitalization by NAFLD status are shown in Fig 1 (panel A and B). The Kaplan-Meier analysis showed that approximately 70% of patients with NAFLD at baseline were readmitted to the hospital at 1 year *vs*. only ~20% of those without NAFLD (*p*<0.0001 for the difference by the log-rank test). Similar results were found for 1-year cardiac re-hospitalization (panel B).

**Fig 2:**
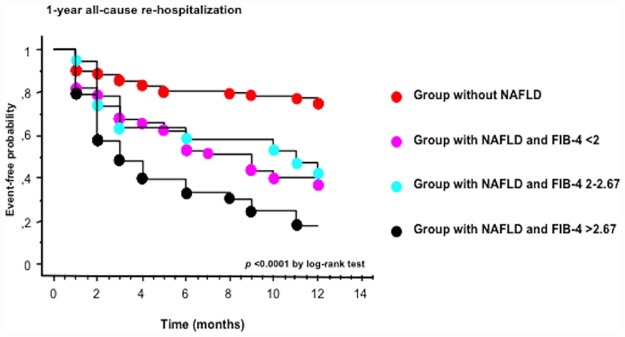
Kaplan-Meier curves. Rates of 1-year all-cause re-hospitalization in hospitalized patients with acute HF stratified by baseline NAFLD status and FIB-4 score The FIB-4 score was used to categorize the severity of advanced liver fibrosis in patients with NAFLD. *P*<0.0001 for the difference by the log-rank test.

As shown in Fig 2, the Kaplan-Meier curves for the rates of 1-year all-cause re-hospitalization showed that the rate of this endpoint was higher in patients with ultrasound-diagnosed NAFLD and high FIB-4 score (FIB-4 >2.67; a marker of advanced NAFLD fibrosis) as compared to other subgroups of patients with normal or intermediate FIB4 scores or those without NAFLD (*p*<0.0001 by the log-rank test). Almost identical results were found for 1-year cardiac re-hospitalization (data not shown). Similar results were also observed when we used other non-invasive fibrosis scores to identify/exclude advanced NAFLD fibrosis, such as the APRI index or the NAFLD fibrosis score (in this latter case, however, the number of patients with available data for calculating the NAFLD fibrosis score was smaller due to the lack of extensive measurement of serum albumin concentrations) (data not shown). However, these results should be interpreted with some caution because all of these non-invasive fibrosis markers have not been sufficiently validated in a non-NAFLD population.

Table 2 shows the effect of adjustment for multiple potential confounders on the association between NAFLD and the risk of 1-year all-cause or cardiac re-hospitalization. In univariable regression analyses (unadjusted model), NAFLD was associated with an approximately 3.5-fold increased risk of all-cause re-hospitalization and with an approximately 5.9-fold increased risk of cardiac re-hospitalization. After adjusting for age, sex and hospital ward (model 1), NAFLD maintained a strong association with 1-year re-hospitalization. The strength of this association was not attenuated after further adjustment for obesity, diabetes, CHD, past history of HF, LV-ejection fraction, eGFR_CKD-EPI_ and plasma NT-proBNP levels (model 2). Finally, additional adjustment for circulating levels of sodium and GGT did not appreciably weaken the association between NAFLD and risk of 1-year re-hospitalization (model 3). Of note, in regression model 3 other independent predictors of increased 1-year all-cause or cardiac re-hospitalization rates, together with NAFLD, were higher baseline levels of plasma NT-proBNP and GGT.

**Table 2:** Cox regression analyses–Associations between NAFLD and risk of 1-year all-cause or cardiac re-hospitalization rates in hospitalized patients with acute HF at baseline.

Cox Hazard Models	Hazard ratio(s)	95% CI	*p* value
***All-cause re-hospitalization***			
**NAFLD (yes *vs*. no)**			
Unadjusted model	3.50	2.23–5.49	<0.0001
Adjusted model 1	3.65	2.28–5.81	<0.0001
Adjusted model 2	4.60	2.69–7.94	<0.0001
Adjusted model 3	5.01	2.78–9.10	<0.0001
***Cardiac re-hospitalization***			
Unadjusted model	5.86	3.27–10.4	<0.0001
Adjusted model 1	6.24	3.44–11.1	<0.0001
Adjusted model 2	8.76	5.30–16.4	<0.0001
Adjusted model 3	8.05	3.77–15.8	<0.0001

Sample size: *n* = 212 for 1-year all-cause re-hospitalizations and *n* = 187 for 1-year cardiac re-hospitalizations, respectively. Data are expressed as hazard ratios ± 95% confidence intervals (CI) as assessed by either univariable (unadjusted) or multivariable Cox hazard models.

Other covariates included in the three multivariable regression models, together with NAFLD, were as follows: *model* 1: age, sex and hospital ward (General Medicine *vs*. Geriatrics); *model* 2: age, sex, hospital ward, past history of HF, diabetes, CHD, obesity (i.e., BMI ≥30 kg/m^2^), eGFR_CKD-EPI_, LV-ejection fraction and plasma NT-proBNP; *model* 3: adjustment for the same variables included in model 2 *plus* serum sodium and GGT levels.

Interestingly, NAFLD remained significantly associated with higher 1-year re-hospitalization rates from all causes (model 3: adjusted-HR 7.02, 95% CI 3.6–13.5) and from cardiac causes (model 3: adjusted-HR 13.1, 95% CI 5.2–31.6) even after excluding patients with re-hospitalization in the early post-discharge period, *i*.*e*., re-hospitalizations at 1 month (*n* = 24).

**Fig 3:**
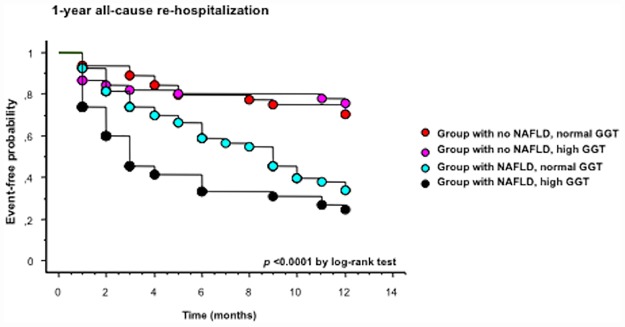
Kaplan-Meier curves. Rates of 1-year all-cause re-hospitalization in hospitalized patients with acute HF simultaneously stratified by baseline NAFLD status and serum gamma-glutamyltransferase (GGT) concentrations (*i*.*e*., high or normal GGT according to its median value ≤46 *vs*. >46 U/l). *P*<0.0001 for the difference by the log-rank test.

Fig 3 shows the cumulative proportions of patients with all-cause re-hospitalization at 1 year after simultaneous stratification by ultrasound-diagnosed NAFLD status and serum GGT levels (*i*.*e*., high GGT >46 U/l or normal GGT ≤46 U/l; this cut-off corresponds to the median value of serum GGT in the whole cohort of patients). The Kaplan-Meier analysis showed that the risk of 1-year all-cause re-hospitalization was greatest in patients with NAFLD and high GGT (approximately 75% of these patients were readmitted to the hospital at 1 year), intermediate in those with NAFLD alone (approximately 65% of these patients were readmitted to the hospital at 1 year) and lowest in those without NAFLD, irrespective of their GGT levels. In particular, the risk of 1-year re-hospitalization was similar for patients without NAFLD with normal GGT and those with high GGT alone (approximately 25% of these patients were readmitted to the hospital at 1 year). Almost identical results were found for 1-year cardiac re-hospitalization (data not shown).

We undertook other sensitivity analyses (subgroup analyses) to evaluate the robustness of our findings. Notably, the association between NAFLD and 1-year all-cause re-hospitalization was consistently demonstrated in all subgroups examined. In particular, we found that there were significant age-adjusted associations between NAFLD and 1-year all-cause re-hospitalization rates in both men (HR 3.12, 95% CI 1.5–6.7) and women (HR 4.16, 95% CI 2.3–7.7), in those admitted to the hospital ward of General Medicine (HR 3.03, 95% CI 1.7–5.3) or Geriatrics (HR 4.5, 95% CI 2.1–10), in those with (HR 3.91, 95% CI 1.2–12.5) and without (HR 3.28, 95% CI 2.0–5.4) past history of HF, in those with (HR 3.23, 95% CI 1.5–7.1) and without (HR 3.70, 95% CI 2.1–6.7) pre-existing CHD, in those with (HR 8.33, 95% CI 2.6–25) and without (HR 2.95, 95% CI 1.7–5.1) established diabetes, in those with (HR 4.33, 95% CI 2.1–8.5) and without (HR 3.95, 95% CI 1.8–5.6) obesity, in those treated with (HR 3.71, 95% CI 1.8–7.7) or without (HR 3.33, 95% CI 1.9–5.9) spironolactone, in those with higher (>1000 pg/ml: HR 5.26, 95% CI 2.6–10) or lower NT-proBNP (≤1000 pg/ml: HR 3.13, 95% CI 1.7–5.9), and in those with LV-ejection fraction below 40% (≤40%: HR 7.15, 95% CI 2.1–24) or above 40% (>40%: HR 3.23, 95% CI 2.0–5.3). Almost identical results were found for 1-year cardiac re-hospitalization (data not shown).

## Discussion

The novel results of this prospective study are as follows: 1) 1-year re-hospitalizations (most of which were due to worsening HF) occurred in 46.7% of patients, who were discharged after their first acute HF admission; 2) the prevalence of ultrasound-diagnosed NAFLD at baseline was approximately 2.5-fold higher in patients with re-hospitalization at follow-up than in those without; 3) patients with NAFLD had remarkably higher rates of 1-year all-cause and cardiac re-hospitalization compared to their counterparts without NAFLD; both event rates were particularly increased in NAFLD patients with advanced hepatic fibrosis (as estimated by the FIB4 score or other non-invasive fibrosis scores); and 4) NAFLD and its severity were independently associated with substantially increased rates of 1-year all-cause and cardiac re-hospitalization. Interestingly, these findings were consistent in all subgroups evaluated, including also in those with and without established diabetes or prior CHD, in those with higher or lower plasma NT-proBNP, and in those with preserved or reduced LV-ejection fraction at baseline.

To our knowledge, this is the largest prospective study aimed at examining the prognostic value of NAFLD *per se* in predicting 1-year all-cause and cardiac re-hospitalization rates in elderly patients admitted for acute HF. Our results extend those that we recently reported in a pilot study on a smaller sample of 107 elderly patients admitted for acute HF, in which NAFLD was found to be significantly associated with higher 1-year all-cause re-hospitalization rates [11]. All these 107 patients were included in the present study, but the sample size was now almost doubled by including also patients admitted to the Geriatric ward of our hospital over the same period of follow-up. Additionally, in the current study more detailed information was also recorded regarding the severity of hepatic fibrosis in patients with NAFLD (using the FIB-4 score and other non-invasive clinical markers of advanced NAFLD fibrosis) as well as the cardiac and extra-cardiac causes of 1-year re-hospitalization for all patients. From a statistical standpoint, the doubling of the sample size of the study (with a consequent significant increase in the total number of clinical outcomes) allowed the achievement of solid and reliable results both from the subgroup analyses and from the multivariable regression analyses after adjustment for established risk factors and potential confounders.

Rates of 1-year re-hospitalization we observed in this study were comparable to those reported by other investigators in large cohorts of hospitalized patients with acute HF with similar baseline demographic characteristics [2,17–19]. In this study, we also found that higher circulating levels of NT-proBNP and GGT were two independent predictors (along with NAFLD) of higher 1-year all-cause and cardiac re-hospitalization rates. Previous studies reported a strong and independent association between higher NT-proBNP and poor clinical outcomes in patients with acute HF [20]. Similarly, previous studies also reported that the presence of severe HF was frequently associated with increased serum GGT, bilirubin and aminotransferase levels [2,20]. Moreover, in a cohort of ambulatory patients with chronic HF, increased serum GGT levels predicted independently the rates of mortality or heart transplantation over a mean follow-up of 34 months [21].

A possible caveat in interpreting the results of this study is that moderately elevated serum liver enzyme levels may be present in patients with acute HF, possibly due to either the use of some potentially hepato-toxic drugs (such as amiodarone or warfarin) or the coexistence of congestive hepatopathy (*i*.*e*., a condition caused by passive venous congestion of the liver that generally occurs in the setting of chronic cardiac conditions, such as chronic HF, constrictive pericarditis, tricuspid regurgitation or right-sided HF of any cause) [2,22]. However, we believe that the most important strength and the added value of our study was that the diagnosis of NAFLD was based on ultrasonography (and not on abnormal serum liver enzyme levels), which is able to differentiate hepatic steatosis from congestive hepatopathy (mainly through the ultrasonographic evaluation of both caval and supra-hepatic veins) [23]. We cannot, obviously, exclude that some of our patients with ultrasound-diagnosed NAFLD and raised serum liver enzyme levels could also have a coexisting congestive hepatopathy. However, our patients with hepatic steatosis (diagnosed by ultrasonography) exhibited the typical anthropometric and biochemical features of NAFLD [24]. Furthermore, as shown in Fig 3, it is important to underline that the highest risk of 1-year all-cause or cardiac re-hospitalizations was observed in patients with hepatic steatosis and high serum GGT, intermediate in those with hepatic steatosis alone, and the lowest in patients without hepatic steatosis, irrespective of their serum GGT levels. In addition, there were no significant differences in baseline LV-ejection fraction or use of potentially hepato-toxic drugs (such as warfarin and amiodarone) between those with and those without re-hospitalization at follow-up. Finally, the significant association between NAFLD and 1-year re-hospitalization persisted even after excluding those with re-hospitalization in the early post-discharge period (*i*.*e*., patients with a higher likelihood of having more ‘severe’ HF and possibly coexisting congestive hepatopathy).

A number of underlying mechanisms can explain the association between NAFLD and increased 1-year re-hospitalization in patients with acute HF. Convincing evidence indicates that NAFLD, especially in its more advanced forms [non-alcoholic steatohepatitis (NASH) and advanced fibrosis], is not only associated with an increased risk of CHD, but is also strongly associated with functional and structural cardiomyopathy that may lead to the development of HF over time [4,6,10,25,26]. Moreover, NAFLD is also associated with enlarged left atrial volume, and increased risk of atrial fibrillation (a known risk factor of new-onset HF) [27–30]. Finally, clear evidence also indicates that NAFLD, especially NASH with varying amounts of hepatic fibrosis, may exacerbate hepatic/peripheral insulin resistance and causes the release of proinflammatory factors, vasoactive factors and thrombogenic molecules that are important in the development of CHD and other functional, structural and arrhythmic complications of the heart [4,6,25]. It is plausible to assume that one of the most important reasons why patients with progressive NAFLD have recurrent HF over time could be largely due to worsening CHD. This assumption may be also true in other different disease models in which there is a high prevalence of hepatic steatosis, such as the chronic infections due to hepatitis C virus (HCV) or human immunodeficiency virus (HIV). Indeed, there is now accumulating evidence reinforcing the assertion that the presence of fatty/inflamed/fibrotic liver is a shared important determinant for the development of CHD and other cardiac complications in patients with HCV or HIV [31].

Our study has some limitations that should be mentioned. Firstly, this study is limited by its single-center, observational design, which limits our ability to establish the causality of the observed associations. Secondly, although our statistical models were extensive, unmeasured confounding factors might partially explain the observed associations. Thirdly, the diagnosis of NAFLD was based on ultrasonography and exclusion of secondary causes of chronic liver disease but was not confirmed by liver biopsy, which is considered as the reference standard for diagnosing and staging NAFLD [32]. However, we believe that it would have been hazardous to perform liver biopsies for these elderly HF patients with normal or only moderately elevated serum liver enzymes. Indeed, ultrasonography enables a reliable and accurate detection of moderate-to-severe hepatic steatosis compared with liver histology. A recent meta-analysis reported that the overall sensitivity and specificity of ultrasonography for the detection of moderate-to-severe fatty liver, compared to histology, were approximately 85% and 95%, respectively [14]. Finally, the use of non-invasive markers of advanced NAFLD fibrosis (such the FIB-4, APRI or NAFLD fibrosis scores) has not been adequately validated in patients with acute HF or in a general population. That said, future studies in larger cohorts of well-characterized patients with NAFLD (as diagnosed by magnetic resonance-proton density fat fraction and magnetic resonance elastography, which are rapidly being recognized as being as good as liver biopsies) [33,34] are needed to better elucidate whether the severity of NAFLD may differentially affect the risk of all-cause and cardiac re-hospitalization in patients admitted for acute HF.

Despite these limitations, our study has important strengths, including its relatively large sample size, the ultrasonographic diagnosis of NAFLD, the completeness of the dataset, the ability to adjust for multiple clinical risk factors and potential confounding factors, and the exclusion of patients with end-stage renal disease, cancer or cirrhosis.

In conclusion, our results show that NAFLD and its severity—using the FIB-4 score or other non-invasive markers of advanced NAFLD fibrosis—were strongly and independently associated with an increased risk of 1-year all-cause and cardiac re-hospitalization in elderly patients admitted for acute HF. Further prospective studies are needed to corroborate these findings in other independent samples.

## Supporting information

S1 Table Baseline clinical and biochemical characteristics of hospitalized patients with acute HF stratified by the hospital ward. (DOCX)
