# Supplementary material for: Nonalcoholic fatty liver disease and increased risk of 1-year all-cause and cardiac hospital readmissions in elderly patients admitted for acute heart failure
Source: PLoS One. 2017 Mar 13;12(3):e0173398. doi: 10.1371/journal.pone.0173398 (PMC5348001; doi:10.1371/journal.pone.0173398)
Supplement: S1 Table — (DOCX) [file pone.0173398.s001.docx]

**S1 Table.** Baseline clinical and biochemical characteristics of hospitalized patients with acute HF stratified by the hospital ward.

|  | Geriatric ward  (*n*=87) | Medicine ward  (*n*=125) | *p* value |
| --- | --- | --- | --- |
| Male sex (%) | 41.4 | 48.0 | 0.34 |
| Age (years) | 85 ± 6 | 80 ± 10 | <0.001 |
| Body weight (kg) | 73 ± 17 | 79 ± 23 | <0.05 |
| Body mass index (kg/m^2^) | 26.6 ± 5 | 27.9 ± 7 | 0.12 |
| Heart rate (bpm) | 88 ± 24 | 81 ± 19 | <0.01 |
| Systolic blood pressure (mmHg) | 133 ± 21 | 131 ± 22 | 0.48 |
| Diastolic blood pressure (mmHg) | 76 ± 12 | 75 ± 13 | 0.81 |
| Pulse pressure (mmHg) | 58 ± 18 | 56 ± 18 | 0.49 |
| Sodium (mmol/l) | 136 ± 5 | 137 ± 6 | 0.44 |
| Potassium (mmol/l) | 4.2 ± 0.5 | 4.2 ± 0.6 | 0.73 |
| Hemoglobin (g/dl) | 12.1 ± 2 | 12.0 ± 2 | 0.54 |
| White blood cell count (x 10^9^/l) | 8.28 ± 4 | 7.96 ± 3 | 0.48 |
| Platelet count (x 10^9^/l) | 223 ± 81 | 225 ± 76 | 0.89 |
| eGFR_CKD-EPI_ (ml/min/1.73 m^2^) | 50.1 ± 20 | 54.0 ± 24 | 0.18 |
| GGT (U/l) | 42 (19 – 89) | 50 (27 – 87) | 0.86 |
| AST (U/l) | 23 (19 – 28) | 24 (18 – 33) | 0.25 |
| ALT (U/l) | 18 (13 – 26) | 19 (13 – 33) | 0.27 |
| NT-proBNP (pg/ml) | 595 (370 – 1107) | 708 (318 – 1344) | 0.15 |
| Total cholesterol (mmol/l) | 3.79 ± 0.9 | 3.63 ± 0.9 | 0.23 |
| Triglycerides (mmol/l) | 1.0 (0.8 – 1.3) | 0.98 (0.8 – 1.2) | 0.21 |
| LV-ejection fraction (%) | 48.3 ± 14 | 47.8 ± 14 | 0.92 |
| LV-ejection fraction ≤40% (%) | 16.1 | 21.1 | 0.37 |
| Diabetes (%) | 29.9 | 48.2 | <0.05 |
| Chronic obstructive pulmonary disease (%) | 18.4 | 17.6 | 0.88 |
| CHD (%) | 32.2 | 34.4 | 0.74 |
| Stroke (%) | 5.7 | 5.6 | 0.98 |
| Pacemaker or ICD (%) | 25.3 | 17.7 | 0.12 |
| Atrial fibrillation (%) | 64.4 | 55.2 | 0.18 |
| Chronic kidney disease (%) | 29.9 | 39.5 | 0.15 |
| ACE-inhibitors/ARB users (%) | 50.6 | 59.7 | 0.20 |
| Furosemide users (%) | 96.6 | 97.6 | 0.41 |
| Spironolactone users (%) | 25.3 | 43.2 | <0.01 |
| Beta-blocker users (%) | 64.4 | 66.1 | 0.79 |
| Digoxin users (%) | 13.8 | 9.7 | 0.35 |
| Amiodarone users (%) | 3.5 | 2.4 | 0.66 |
| Antiplatelet drug users (%) | 32.2 | 50.0 | <0.05 |
| Oral anticoagulant users (%) | 37.9 | 40.3 | 0.73 |
| Statin users (%) | 29.1 | 21.8 | 0.43 |
| Hospital stay (days) | 15.6 ± 6 | 11.8 ± 6 | <0.001 |
| NAFLD (%) | 42.5 | 57.5 | <0.05 |

Sample size, *n*=212. Data are expressed as means ± SD, medians (IQR) or relative proportions.

Note: Measurements of NT-proBNP and LV-ejection fraction were available only in 206 and 196 patients, respectively.

Abbreviations: ARB, angiotensin receptor blocker; ALT, alanine aminotransferase; AST, aspartate aminotransferase; CHD, coronary heart disease; eGFR, estimated glomerular filtration rate (as estimated by the CKD-EPI equation); GGT, gamma-glutamyltransferase; LV, left ventricular; NAFLD, nonalcoholic fatty liver disease; NT-proBNP, NT pro-brain natriuretic peptide.
